# Investigation of the Optical Nonlinearity for Au Plasmonic Nanoparticles Based on Ion Implantation

**DOI:** 10.3390/nano13192662

**Published:** 2023-09-28

**Authors:** Huiyuan Chu, Hongpei Wang, Yancheng Huang, Hao Dai, Menglu Lv, Ziyang Zhang, Cheng Jiang

**Affiliations:** 1 College of Electronic and Information, Qingdao University, Qingdao 266071, China; chuhuiyuan2021@163.com (H.C.); 18705457002@139.com (Y.H.); daihao0117@163.com (H.D.); lml567817@163.com (M.L.); 2 School of Nano-Tech and Nano-Bionics, University of Science and Technology of China, Hefei 230026, China; hpwang@mail.ustc.edu.cn; 3 Qingdao Yichen Leishuo Technology Co., Ltd., Qingdao 266318, China

**Keywords:** ion implantation, Au nanoparticles, local plasma resonance effect, saturable absorber

## Abstract

The Au ion implantation process has emerged as an effective and simple method to be utilized for the fabrication of opto-electronic materials and devices due to numerous fascinating features of Au nanoparticles such as surface plasmon resonance (SPR), large third-order nonlinearity and a fast response time. In this paper, we describe the fabrication of a novel Au nanoparticle saturable absorber (Au NP-SA) by embedding the Au NPs into a SiO_2_ thin film using the ion implantation process, which shows excellent saturable absorption features due to the localized surface plasmon resonance (LSPR) effect of Au NPs. A stable and high-quality pulsed laser with a repetition rate of 33.3 kHz and a single pulse energy of 11.7 nJ was successfully constructed with the Au NP-SA. Both the stable operation characteristic of the obtained Q-switched pulsed laser and the high repeatability of the fabrication process of the Au NP-SA were demonstrated. In addition, the simple feasibility and maturity of the ion implantation process allow for the plasmonic nanoparticles to be easily integrated into other types of opto-electronic materials and devices to further improve their performance, and shows immense potential for the production of wafer-level products.

## 1. Introduction

Metal nanoparticles (NPs) have attracted tremendous interest due to their unique localized surface plasmon resonance (LSPR), which is a size effect caused by collective carrier oscillations when electromagnetic waves interact with metal NPs or nanostructures [1,2,3]. A strong LSPR has been found in many kinds of materials including noble metals, Al and alkalis. NPs can strongly absorb the photon energy exciting LSPR when light is incident on NPs composed of metals, and the frequency of the incident photons match the vibration frequency of the electrons inside metal NPs. The LSPR can considerably enhance the electric field to enhance light absorption and can also obtain resonance spectra to study the microscopic composition of NPs and their surrounding media, so the effects brought by a nanoscale LSPR have led to extensive research in sensors, spectral analysis, detectors and other fields [4,5,6]. The collective oscillation of free carriers in metal NPs could be coupled with the particular incident light, and the optical field can be effectively observed within a picosecond time scale [7]. A part of the incident light is absorbed, exciting non-hot carrier generation through Landau damping, subsequently relaxing through electron–electron interactions within 100 fs and electron–phonon interactions with a few ps [8]. Due to the excellent third-order nonlinear optical properties of NPs, including an ultra-short recovery time, a large third-order nonlinear coefficient, controllable adjustment parameters, a wide surface plasmon resonance (SPR) absorption range and fast response time [9], metal NPs have been widely applied in the fields of optics, medicine and spectroscopy [10,11,12]. However, the majority of NPs predominantly rely on solution-based or self-organizing techniques [13,14]. Regrettably, controllability, stability and repeatability are significant challenges that severely inhibit the extensive application of NPs. 

Compared to the methods mentioned above, the ion implantation process [15,16] is a relatively mature technology for synthesizing NPs with abundant advantages such as the uniformity of produced NPs. The ion implantation method can achieve large-scale material modification through simple operations, forming new doping systems and obtaining excellent device performance. Therefore, ion implantation stands out as a highly suitable and advanced technique for synthesizing metallic NPs within solid host matrices. With its precise control and compatibility with diverse materials, it offers unparalleled potential for NPs synthesis [17]. The process of ion implantation has a certain degree of flexibility and designability. The materials of ion implantation can be flexibly designed according to the needs, including the selection of substrate materials and implanted ions. The size and distribution of NPs in the material vary with changes in ion implantation parameters. Setting appropriate conditions such as the ion implantation energy, injection dose and ion density can achieve ideal results. Meanwhile, the ion implantation method results have portability, high reliability and repeatability. Compared to the commonly used solution methods for research, ion implantation can obtain stable device results, modify one or more layers of thinner or thicker materials and can be applied in various fields, including lasers, sensors, detectors, etc. It is also expected to achieve large-scale integrated device manufacturing.

Here, the ion implantation method was utilized to fabricate a novel type of Au nanoparticle saturable absorber (Au NP-SA), in which the Au NP-doped SiO_2_ film was grown on a highly reflective mirror via plasma enhanced chemical vapor deposition (PECVD). Based on the Au NP-SA, a stable and high-quality Q-switched pulsed laser with a repetition rate of 33.3 kHz and a single pulse energy of 11.7 nJ was successfully realized. The FDTD (finite-difference time-domain) software (version FDTD2020) is a widely used tool for the simulation of an electromagnetic field. It is based on the FDTD method and can simulate and analyze complex electromagnetic phenomena. The enhancement of the electric field generated by Au NPs was verified through optical simulation using FDTD. Furthermore, the ion implantation process for embedding Au NPs is easily integrated into a wide range of opto-electronic materials and devices, enhancing their performance. This straightforward implementation nature allows for the incorporation into existing fabrication processes, making it a practical choice for producing wafer-level products.

## 2. Materials and Methods

### Au NP-SA Sample Preparation and FDTD Simulation

**Figure 1:**
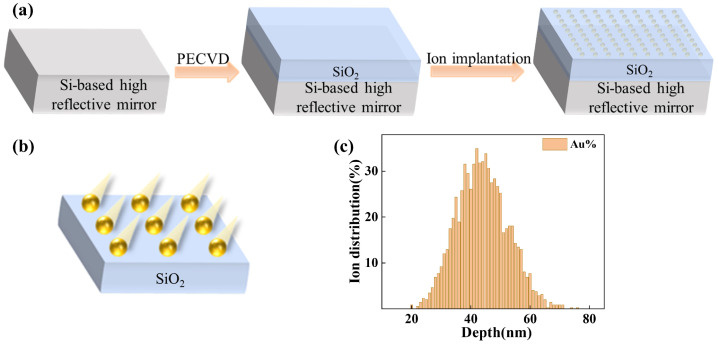
(**a**) Schematic illustration of Au NPs-SA preparation process; (**b**) Au ion implantation; (**c**) Calculated ion distribution by SRIM software (http://www.srim.org/).

The selection of ion implantation materials is of paramount importance due to its direct impact on the material system characteristics of the implantation area. Among various metal NPs, Au NPs exhibit several notable advantages, rendering them highly preferred in numerous research domains and practical applications. Au is biologically inert and non-toxic, making Au NPs highly compatible with living organisms. Furthermore, Au NPs demonstrate exceptional stability and exhibit reduced susceptibility to oxidation when compared to numerous other metal NPs. The inherent stability of Au NPs allows for prolonged storage and utilization without substantial degradation or modifications in their characteristics. Additionally, these NPs display LSPR, enabling robust interactions with light and demonstrating unique optical properties. The schematic illustration of the fabrication process for Au NP-SA is shown in Figure 1a. To simplify the model, we sketch it as a periodic structure. To control the distance of Au ion sputtering and the range of the LSPR effect, a 130 nm thick layer of SiO_2_ film was deposited on the Si-based highly reflective mirror using the PECVD process. Subsequently, Au ion implantation was performed using the FM2000 metal ion implantation machine. The FM2000 machine utilizes an ion beam generated by the MEVVA (Metal Vapor Vacuum Arc) source to perform ion implantation on the device surface. This process allows for the modification of the performance and conduction of surface modification research on ion implanted materials. The high-speed ions are accelerated through an electric field and directed towards the target material. During the incident procedure, these ions collide with the atoms in the target material, losing energy and ultimately becoming embedded within the material. When the concentration of incident atoms (injection dose) exceeds the solid solubility of the substrate, dispersed particles start forming clusters, leading to the formation of metal NPs. The ion implantation method can provide a matrix with a maximum filling factor that is higher than the equilibrium solubility threshold for metal NPs implanted into the dielectric, and can be used to form all kinds of metal particles. Meanwhile, the profile for implanted materials agrees with the size distribution of NPs, and the concentration of metal begins to monotonically decrease near the surface [18,19]. As shown in Figure 1b, the ion injection process was conducted under vacuum condition. The fabrication of the Au NP-SA sample involved attaching the structured sample onto a target plate and placing it inside an ion implantation vacuum chamber. An Au target plate was selected, and the injection angle was set to positive incidence, corresponding to an incident angle of 0°. The beam density utilized for the injection process was 4 μA/cm^2^. Au NP-SA sample can be obtained by implanting Au ions with an injection energy of 100 keV and an injection dose of 1 × 10^17^ ions/cm^2^. To demonstrate clearly, ion distribution was calculated using the Stopping and Range of Ions in Matter (SRIM) software (http://www.srim.org/) [20,21], which can be used to simulate and calculate the approximate distribution of Au ion concentration within the silica layer, as shown in Figure 1c. Following the implantation of Au ions, these discrete ions aggregate to form Au NPs in medium [22,23]. The distribution of Au NPs in silica exhibits a Gaussian distribution, with the majority of the NPs located approximately 30–60 nm beneath the surface of the silica.

**Figure 2:**
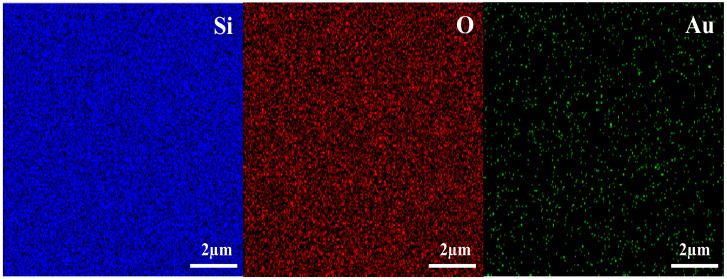
EDS elemental content of the Au NP-SA sample.

The composition and contents of the material microregions in the Au NP-SA sample were further analyzed using energy dispersive spectroscopy (EDS), as depicted in Figure 2. The EDS spectrum clearly shows the elements present in the sample, including Si, O and Au, which can strongly prove that the sample has undergone the Au ion implantation process. In addition, it can be clearly seen from Figure 2 that the distribution of all elements is relatively uniform, and no particularly obvious voids were found, providing strong evidence for the uniform distribution of the Au element within the silica layer. At the same time, these findings suggest the potential of ion implantation as a method for producing wafer-level products with excellent uniformity, reliability and efficiency in synthesizing NPs, which can be used for the large-scale production of material modification or device performance improvement.

**Figure 3:**
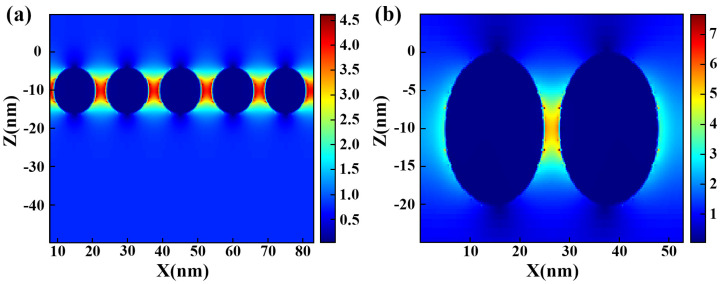
(**a**) Simulation of field strength between Au NPs. (**b**) Simulation of field strength between two Au NPs.

Finite-difference time-domain (FDTD) simulation is a powerful technique for investigating the interaction between electromagnetic waves and nanostructures. By using the numerical solution of Maxwell’s equation, FDTD simulation enables the accurate analysis of the electric field distribution and enhancement effects generated around Au NPs. By combining the FDTD method to simulate the local electric field distribution of Au NPs, the local electric field enhancement effect of Au NPs can be more vividly and reliably observed, further verifying the effect of LSPR on the changes in the electric field intensity due to Au ion implantation (Figure 3). In the simulation, periodic boundary conditions are set in the X and Y directions, while a Perfectly Matched Layer (PML) is employed in the Z direction to minimize reflection.

To ensure accurate measurements and observations, it is important to maintain appropriate lighting and monitor conditions. To verify the LSPR effect of embedded Au NPs, an Au NP distribution was constructed to obtain electric field enhancement. A small-scale Au NP model was used for the simulation, and the Au NPs were simulated as spherical structures. In Figure 3a, a depiction is provided of uniformly dispersed Au NPs within SiO_2_ medium, where the dark blue spheres represent the Au NPs. These graphs in different colors can represent the corresponding electric field intensity. The color change can intuitively help us understand the strength of the electric field at different positions through the whole structure. The corresponding electric field enhancement can be determined by the numerical values corresponding to the colors on the right side of the simulation diagram. The red color indicates a higher magnitude of electric field strength. It can be clearly seen that the electric field intensity in this area was effectively enhanced due to the presence of Au NPs. The incident light has a wavelength close to 1550 nm. Notably, the electric field intensity at two Au NPs is observed to be higher compared to other locations, indicating the occurrence of a strong LSPR effect between Au NPs under the irradiation of the incident light of the corresponding resonance wavelength, resulting in a remarkable enhancement in the electric field intensity. Figure 3b presents a further analysis of the electric field distribution between the two adjacent Au NPs using FDTD simulation. The simulations serve to validate the resonant electric field enhancement effect generated by the Au NPs. As the absorption of light is directly proportional to the square of the electric field intensity, the light absorption capability of the Au NPs is greatly enhanced. These findings highlight the crucial role of LSPR in enhancing the absorption of light by Au NPs.

**Figure 4:**
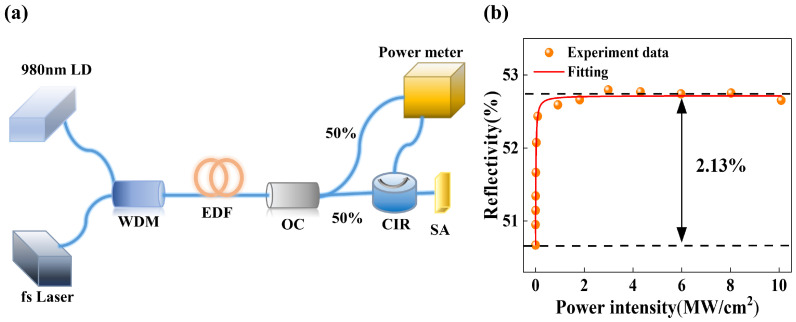
(**a**) Schematic illustration of the balanced twin-detector measurement. (**b**) Nonlinear saturable absorption curves of the Au NP-SA.

A self-made two-arm nonlinear test system was utilized to evaluate the nonlinear optical properties of the Au NP-SA sample, as shown in Figure 4a. This system allows for the accurate measurement and analysis of the nonlinear behavior of the sample. The nonlinear system was constructed using a femtosecond laser seed source, a 980 nm pump laser diode (LD), a 980 nm/1550 nm wavelength division multiplexer (WDM), an 11 m erbium-doped fiber (EDF), a 50:50 optical coupler (OC), a circulator (CIR) and a power meter. The femtosecond laser seed source employed in the system is a mode-locked laser developed by our laboratory. It operates at a central wavelength of 1560 nm, with a pulse width of 820 fs, and a repetition rate of 13 MHz. The OC divided the output laser and pump source into two paths after passing through WDM and EDF. One part of optical power is directed into the Au NP-SA sample, while the other part of optical power serves as a reference value [24]. By incorporating a reference arm next to the Au NPs-SA sample, comparative analysis can be performed, facilitating accurate measurements and ensuring the reliability and significance of the obtained results. The nonlinear absorption curve of the Au NP-SA sample is shown in Figure 4b. After conducting the test and fitting the data using Equation (1) [25], the modulation depth of the Au NPs-SA sample was obtained about 2.13%, which demonstrates the excellent nonlinear absorption performance of the Au NP-SA.

(1) $$R=1-\Delta R\times \exp(-P/P_{sat})-R_{ns}$$

where *R* is the reflectivity, Δ*R* is the modulation depth, *P*_sat_ is the incident saturation optical power and *R*_ns_ is the unsaturated loss. The injected Au NPs serve as local plasmon materials, which can enhance the local electromagnetic field and promote the interaction between light and matter. The LSPR effect greatly enhances the interaction between the incident light and the metal plasma NPs, resulting in a higher third-order optical nonlinear magnetization rate and ultra-fast nonlinear response time [15,26,27,28]. Exposure of Au NPs to incident light induce the inherent LSPR effect, resulting in pronounced surface charge density oscillations on the metal NPs. Due to their diminutive size, the scattering effect of the Au NPs is negligible, resulting in substantial absorption of the incident light by the Au NPs instead. The electrons of Au NPs are polarized under the external light field, which enhances the electric field around them and produces resonance-enhanced absorption, showing a high third-order nonlinear effect. These NPs can be utilized to achieve significant enhancement in optical properties and exhibit promising functionalities in various technological advancements.

## 3. Results and Discussion

The saturable absorption characteristic is intricately linked to the nonlinear optics of the SA materials. By modulating the nonlinear light absorption behavior of the SA materials, various applications such as light intensity modulation, beam modulation and the precise control of the light pulse generation can be achieved. There are many sorts of high-performance SAs including semiconductor saturable absorber mirrors (SESAMs) [29], topological insulators [30], black phosphorus [31], transition metal sulfides [32], graphene [33,34], etc. However, there are also some inherent limitations of them; for example, the design and fabrication of the SESAM is complex and cost-intensive, while preparing a certain thickness of the saturable absorber for the topological insulator is challenging. Black phosphorus is prone to oxidation, and transition metal sulfide can encounter issues related to defect energy levels. Graphene has a low damage threshold and is susceptible to generating defects [35,36]. Here, we successfully developed a highly efficient passive Q-switched laser [32,37,38] by harnessing the exceptional nonlinear optical features of the Au NPs. In the operation state, the free continuous light from the laser cavity is incident on the Au NP-SA. When the energy of the incident light is strong enough, the saturable absorber begins to absorb the light, causing the charge carriers on the valence band to be excited to the conduction band. As the light intensity increases, the number of electrons on the conduction band gradually increases, and the absorption of light by saturable absorbers reaches saturation, allowing for light pulses to pass through without loss. Consequently, the incident light is redirected back into the laser cavity via the reflective mirror. When the pump power exceeds the established threshold, a dynamic equilibrium is achieved within the cavity, resulting in the generation of a pulse output [39].

**Figure 5:**
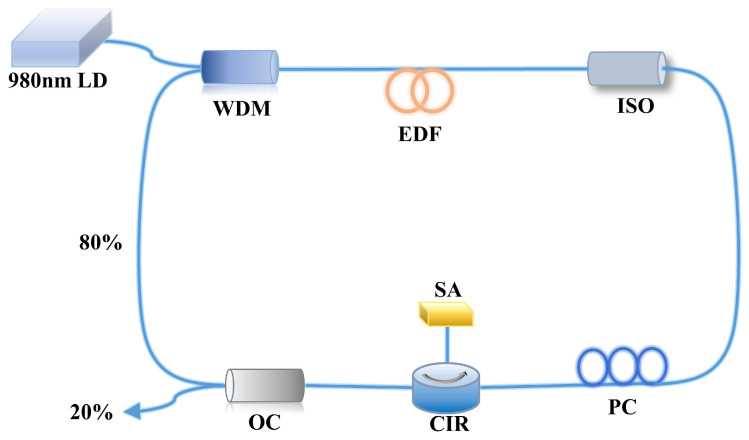
Passive Q-switched laser schematic diagram of the experimental setup.

The Au NPs are used as SA materials of the ring cavity passive Q-switched laser to further verify the excellent characteristics of the sample fabricated via the ion implantation process. The passive Q-switched laser consists of an LD with a central wavelength of 980 nm, a 980 nm/1550 nm WDM, a 4 m EDF, a polarization isolator (ISO), a polarization controller (PC), CIR, OC and the Au NP-SA sample. These devices are interconnected through single-mode optical fibers according to the optical path system diagram shown in Figure 5. The laser pump source emits continuous light with a central wavelength of 980 nm, which is subsequently coupled into the annular cavity through the WDM. The Er^3+^ that is present in the EDF converts the continuous light at 980 nm to steady light at 1550 nm. The protection of the pump laser through unidirectional light transmission in the fiber system is facilitated by the ISO. The PC is then employed to adjust the polarization state of the light, after which the light enters the CIR and irradiates onto the SA. With the presence of the highly reflective mirror, the light re-enters the ring cavity. Ultimately, a fraction of 20% of the light is emitted through the OC exclusively for testing purposes, while the remaining 80% is effectively conveyed back into the ring cavity via the WDM.

**Figure 6:**
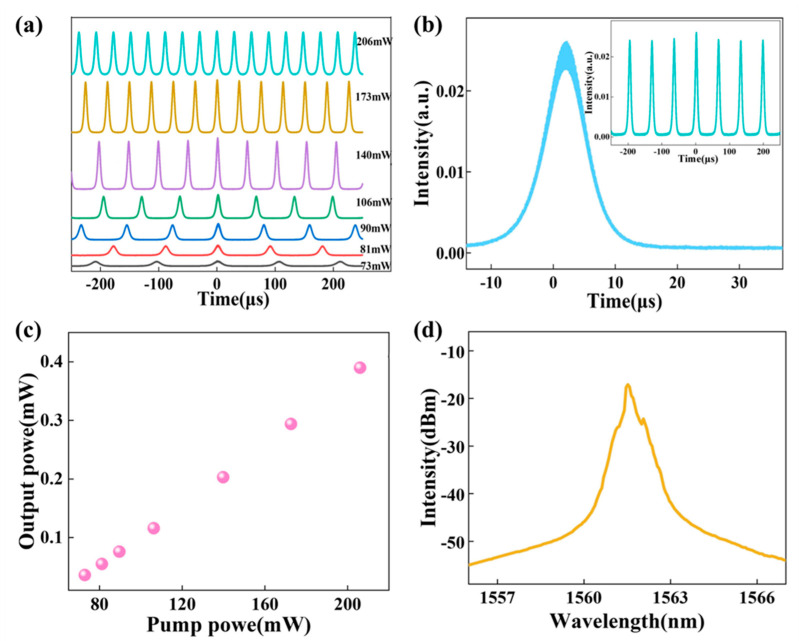
Schematic diagram of passive Q-switched fiber laser. (**a**) Q-switched pulse sequence at different power. (**b**) Single pulse profile and Q-switched pulse sequence. (**c**) Variation curve of output power with pump power. (**d**) Q-switched laser output spectrum.

The Au NP-SA sample was placed in the constructed fiber laser circuit, and the time-domain pulse sequence, output spectrum and output optical power of the pulse laser were characterized using a digital oscilloscope (Keysight DSOS054A), a spectrum analyzer (Anrits MS9740A) and a power meter to test the Q-switched output characteristics, respectively. The laser remains in a continuous wave state without the Au NP-SA, while a transition occurs after the Au NP-SA is introduced into the ring cavity, enabling the laser to operate in a passive Q-switched state. The electrons of the plasmonic Au NPs close to the Fermi level can be easily excited to a virtual high-energy surface plasmon (sp) state, resulting in the generation of hot electrons. Subsequently, these hot electrons rapidly thermalize to establish a hot Fermi–Dirac distribution. With the incident light intensity increasing, the sp state reaches saturation, leading to the blocking of additional optical absorption. Concurrently, the hot electrons cool down via the Auger heating effect and undergo electron transfer processes to return to the Fermi level. In a state of dynamic equilibrium, the plasmonic Au NPs can periodically modulate intracavity loss and induce a saturable absorption effect [40,41]. The considerable third-order nonlinearity exhibited by NPs facilitates a direct positive correlation between the nonlinear absorption and third-order nonlinear coefficient [42,43,44]. The remarkable capability of the Au NPs to modulate light absorption provides a straightforward way to achieve a Q-switched state. The results of the oscilloscope test are presented in Figure 6. Figure 6a demonstrates the stable Q-switched operation of the Au NPs-SA sample within a pump power range of 73 mW to 206 mW. Figure 6b displays the profile of the Q-switched mono pulse at a pump power of 106 mW, with the inset image showcasing the pulse sequence corresponding to the current power. The power variation curve with respect to the pump power is depicted in Figure 6c, indicating a nearly linear increase in the output power up to approximately 0.39 mW as the pump power rises from 73 mW to 206 mW. Throughout this range, a stable Q-switched state is attainable. Furthermore, Figure 6d exhibits the emission spectrum of the Au NP-SA, showcasing a central wavelength at approximately 1562 nm.

**Figure 7:**
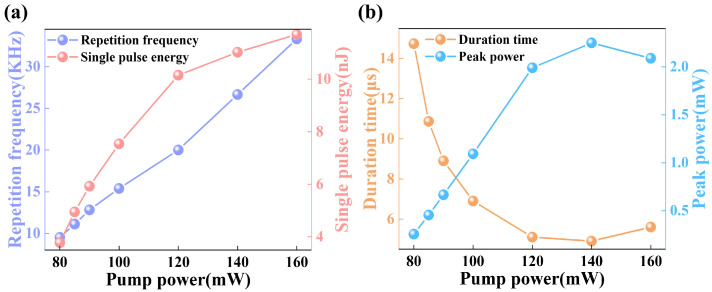
Output characteristics of the Q-switched laser based on the Au NP-SA. (**a**) Variation curves of the repetition frequency and single-pulse energy with pump power. (**b**) Variation curves of the duration time and peak power with pump power.

Figure 7a shows the variation curves of the output repetition frequency and single pulse energy as a function of the pump power. As the pump power increases from 73 mW to 206 mW, the pulse duration gradually decreases, while the repetition frequency steadily increases from 9.5 kHz to 33.3 kHz. As the pump power increases, the pulse width decreases, and the repetition frequency increases, exhibiting typical characteristics of passive Q-switched lasers. As the pump power increases, the single pulse energy of the Q-switched laser output also shows an increasing trend and can reach a maximum of 11.7 nJ. The output waveform is roughly observed using an oscilloscope, and the Q-switched pulse width achieved by the Au NP-SA is of the μs level, which cannot be directly observed using an autocorrelator. Therefore, the pulse widths of different powers were obtained using the Gaussian fitting method, and Figure 7b shows the variation curves of the pulse width and peak power with the pump power. The output pulses of the Au NP-SA-based Q-switched laser have a relatively small pulse width. The minimum pulse width is 5.6 μs, which indicates that the sample can compress the energy into a short time for the laser output. Of course, the pulse duration can be further reduced by optimizing the structure of the laser cavity, for example, by reducing the length of the laser cavity. Figure 7b also shows the variation of the single-pulse peak power with the pump power, which can reach 2.25 mW.

The above analyses effectively demonstrate the remarkable nonlinear characteristics of the Au NP-SA formed through ion implantation. At the incidence of the light, the high-intensity electric field triggers a pronounced polarization effect within the NPs, resulting in a nonlinear response. The substantial third-order nonlinearity of the Au NPs facilitates efficient nonlinear absorption and modulation of the incident light, enabling effective control over the laser output to generate short and high-intensity pulses. The distinctive properties of Au NPs make them highly desirable as saturable absorbers for the easy implementation of Q-switched lasers. Of course, in other optical device fields such as photodetectors, optical sensors, optical switches, etc., ion implantation can also be used to form metal NPs for device optimization or integration. By utilizing the local electric field enhancement brought by Au NPs to enhance light absorption and nonlinear characteristics, the overall performance of the device can be further improved and applied in more fields. An additional notable advantage of embedding Au NPs in devices is their inherent stability, which ensures the long-term reliability and durability of Au NPs formed by ion implantation under various conditions. Such stability is of paramount importance for the integration of Au NPs into various photonic systems. 

## 4. Conclusions

We successfully demonstrated that the novel Au NP-SA, prepared using the ion implantation method, exhibits exceptional nonlinear properties. The LSPR effect between the Au NPs enables the sample to exhibit remarkable performance in the nonlinear test, with a modulation depth reaching up to 2.13%. Therefore, the device can be effectively utilized as an SA optical device to achieve a stable Q-switched pulse laser output. The simplicity and robust reproducibility of the ion implantation method for preparing Au NPs make it highly suitable for long-term usage. Ion implantation demonstrates convenient integration capabilities with other devices, facilitating the fabrication of photonic integrated devices. This integration significantly enhances the overall performance of the devices and creates opportunities for large-scale production. Consequently, it expands the scope of ion implantation applications and the advancement of Au NPs, offering a novel approach to the preparation of high-performance devices.

## Data Availability

The data presented in this study are available upon request from the corresponding authors.
